# The diagnostic accuracy of an intelligent and automated fundus disease image assessment system with lesion quantitative function (SmartEye) in diabetic patients

**DOI:** 10.1186/s12886-019-1196-9

**Published:** 2019-08-14

**Authors:** Yi Xu, Yongyi Wang, Bin Liu, Lin Tang, Liangqing Lv, Xin Ke, Saiguang Ling, Lina Lu, Haidong Zou

**Affiliations:** 1 Shanghai Eye Disease Prevention & Treatment Center / Shanghai Eye Hospital, Shanghai Key Laboratory of Ocular Fundus Diseases; Shanghai General Hospital, Shanghai Engineering Center for Visual Science and Photomedicine, 380 Kangding Road, Shanghai, 200040 China; 2 Shenzhen Nanshan Center for Chronic Disease Control, No. 7, Huaming Road, Nanshan District, Shenzhen, 518064 China; 3 Shanghai Radio Equipment Research Institute, No. 203, Liping Road, Shanghai, 200090 China; 4 EVision technology (Beijing) Co. LTD., No.26, Shangdixinxi Road, Haidian District, Beijing, 100085 China

**Keywords:** Diabetic retinopathy, Screening, Digital imaging processing, Lesion quantification, Epidemiology

## Abstract

**Background:**

With the diabetes mellitus (DM) prevalence increasing annually, the human grading of retinal images to evaluate DR has posed a substantial burden worldwide. SmartEye is a recently developed fundus image processing and analysis system with lesion quantification function for DR screening. It is sensitive to the lesion area and can automatically identify the lesion position and size. We reported the diabetic retinopathy (DR) grading results of SmartEye versus ophthalmologists in analyzing images captured with non-mydriatic fundus cameras in community healthcare centers, as well as DR lesion quantitative analysis results on different disease stages.

**Methods:**

This is a cross-sectional study. All the fundus images were collected from the Shanghai Diabetic Eye Study in Diabetics (SDES) program from Apr 2016 to Aug 2017. 19,904 fundus images were acquired from 6013 diabetic patients. The grading results of ophthalmologists and SmartEye are compared. Lesion quantification of several images at different DR stages is also presented.

**Results:**

The sensitivity for diagnosing no DR, mild NPDR (non-proliferative diabetic retinopathy), moderate NPDR, severe NPDR, PDR (proliferative diabetic retinopathy) are 86.19, 83.18, 88.64, 89.59, and 85.02%. The specificity are 63.07, 70.96, 64.16, 70.38, and 74.79%, respectively. The AUC are PDR, 0.80 (0.79, 0.81); severe NPDR, 0.80 (0.79, 0.80); moderate NPDR, 0.77 (0.76, 0.77); and mild NPDR, 0.78 (0.77, 0.79). Lesion quantification results showed that the total hemorrhage area, maximum hemorrhage area, total exudation area, and maximum exudation area increase with DR severity.

**Conclusions:**

SmartEye has a high diagnostic accuracy in DR screening program using non-mydriatic fundus cameras. SmartEye quantitative analysis may be an innovative and promising method of DR diagnosis and grading.

## Background

In recent decades, the number of diabetes mellitus (DM) patients worldwide has grown rapidly. The global prevalence of DM patients is estimated to increase from 2.8 to 4.4% between 2000 and 2030 [1], including an increase of 20% in industrialized nations and of 69% in developing countries [2]. Diabetic retinopathy (DR), the most frequent microvascular complication in DM, has become the leading cause of blindness in adults of working age [3]. The incidence of DR increases with disease duration [4]. In patients with type 2 diabetes with a duration of more than 20 years, the prevalence of DR increases to 60% [5]. DR is characterized by retinal microaneurysms, hemorrhages, lipid exudation, vascular closure, and neovascularization [6].

With the prevalence of DM increasing annually, human grading of retinal images to assess DR poses a large burden worldwide [7]. Evaluating DR through remote retinal image reading lowers the barriers to eye examination and provides generalized eye healthcare opportunities to DM patients who might not have the opportunity to receive regular eye healthcare [8]. Some example programs include the UK National Health Service Diabetic Eye Screening Program (NHS DESP) [9] and the Indian Health Service (IHS)-Joslin Vision Network (JVN) in the United States [10]. Although both were effective, they depended on trained human graders and substantial investments.

Digital imaging and imaging processing have contributed to the broad use of image analysis techniques in ophthalmology [11, 12]. Traditional image reading is based solely on doctors’ recognition of ocular fundus images. This process not only is time consuming for doctors but also relies on doctors’ skill and experience, thus greatly limiting the efficiency of treatment of fundus diseases [13, 14]. Therefore, to perform feature analysis of fundus images, we have recently developed a fundus image processing and analysis system for DR screening (SmartEye, version 3.0). SmartEye is based on computer vision technology and is sensitive to the lesion area. This system can identify the lesion position automatically and can aid in determining disease stage by marking the size of the lesion area. SmartEye greatly decreases doctors’ burden of reading fundus images, thus affording them more time to focus on disease treatment. With the help of SmartEye, DR screening would also be feasible in areas lacking ophthalmologists. The present study examines the screening performance of SmartEye and compares the results of automatic grading and human graders.

## Methods

### Study design and participants

This study was approved by the Ethics Committee of the Shanghai Eye Disease Prevention & Treatment Center. No personal information could be recognized or be disclosed from the imaged used in this study. This study was carried out in accordance with the Declaration of Helsinki. The object of the study was to evaluate the screening performance and accuracy of SmartEye. During the DR screening program (Shanghai Diabetic Eye Study in Diabetics (SDES), NCT03579797) in Shanghai from Apr 2016 to Aug 2017, 19,904 fundus images from 6013 patients were acquired. The screening program was organized by the Shanghai Eye Disease Prevention & Treatment Center (SEDPTC). All retinal images were collected with a non-mydriatic fundus camera (NW 400, Topcon, Japan) by community healthcare professionals who had been trained by fundus disease experts in SEDPTC. Two fundus images centered on the macula and on the optic disc were taken from each eye of each DM patient. For grading patients’ images, three ophthalmologists who are retinal specialists were invited to decide whether the images were qualified for grading and then graded the fundus images independently. The image grading standard is referred to the proposed international clinical diabetic retinopathy and diabetic macular edema disease severity scales [15]. Once the independent grading were finished, the grading ophthalmologists had a consensus meeting to discuss images without initial agreement on the image quality or DR stages until an agreement was reached.

### Automated retinal image analysis systems

The automatic DR screening system (SmartEye, version 3.0) identifies DR through recognizing and analyzing lesions in patient fundus images and comparing them against images of classical DR lesions.

The fundus images was firstly processed including drying and normalization. The purpose of normalization is to ensure that the color, brightness, and exposure of images are in the same gray value range to improve the feature extraction accuracy of mass images. Then based on the global gray value analysis, we performed grayscale threshold segmentation with the features of color, brightness, contrast, and combined with mathematical morphology to extract lesions of fundus, such as microaneurysm, hemorrhage, and exudation. To obtain correct information from different fundus images, the lightness, brightness, and saturation of the fundus images are normalized by referring to a standard image, and the vascular boundary is extracted according to intensity threshold separation and supervised classification based on color characteristics. Then the features of hemorrhagic points and microaneurysms in the fundus images are identified comprehensively with mathematical morphology and a support vector machine. On the basis of the feature integration theory of the human visual attention mechanism and Bayesian theory, the shape, color, and correlation of lesions are analyzed to discriminate the type of disease. We utilize the classification method of decision tree to analyze the data characteristics of different grades of DR, and generate DR classification rules based on the idea of multiple regression. Finally disease staging is performed according to the staging standards for DR. If characteristic lesions are found in the fundus images, suspicion of DR is determined. Another function of SmartEye is quantifying the lesion of fundus based on pixels. SmartEye consists of the following modules:

### Image pre-processing

**Fig. 1:**
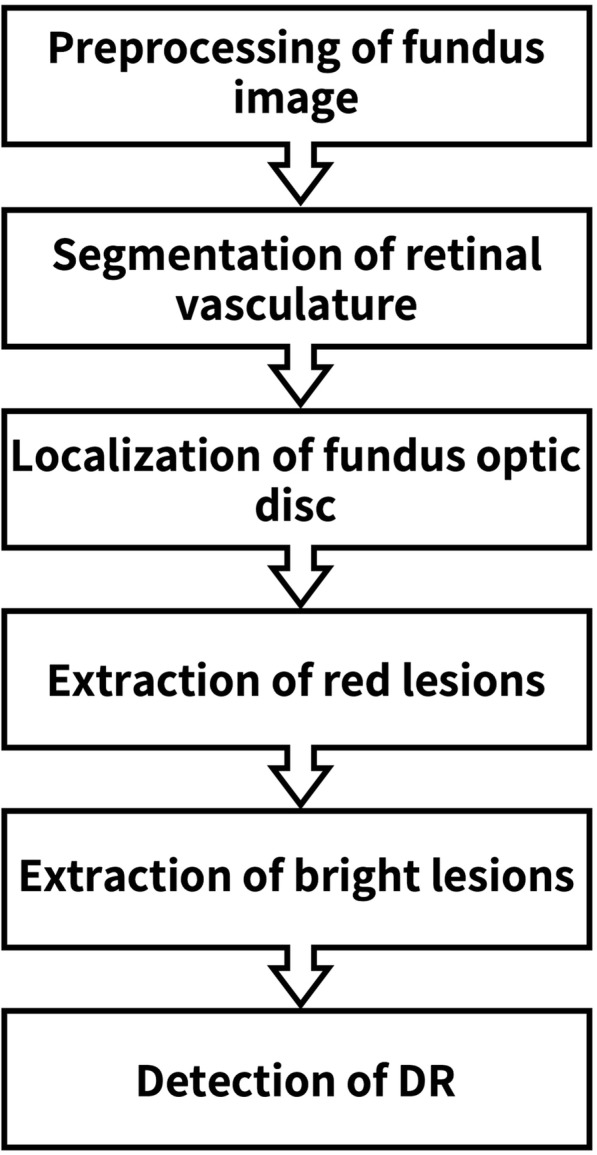
The image processing and analysis flow diagram of SmartEye

**Fig. 2:**
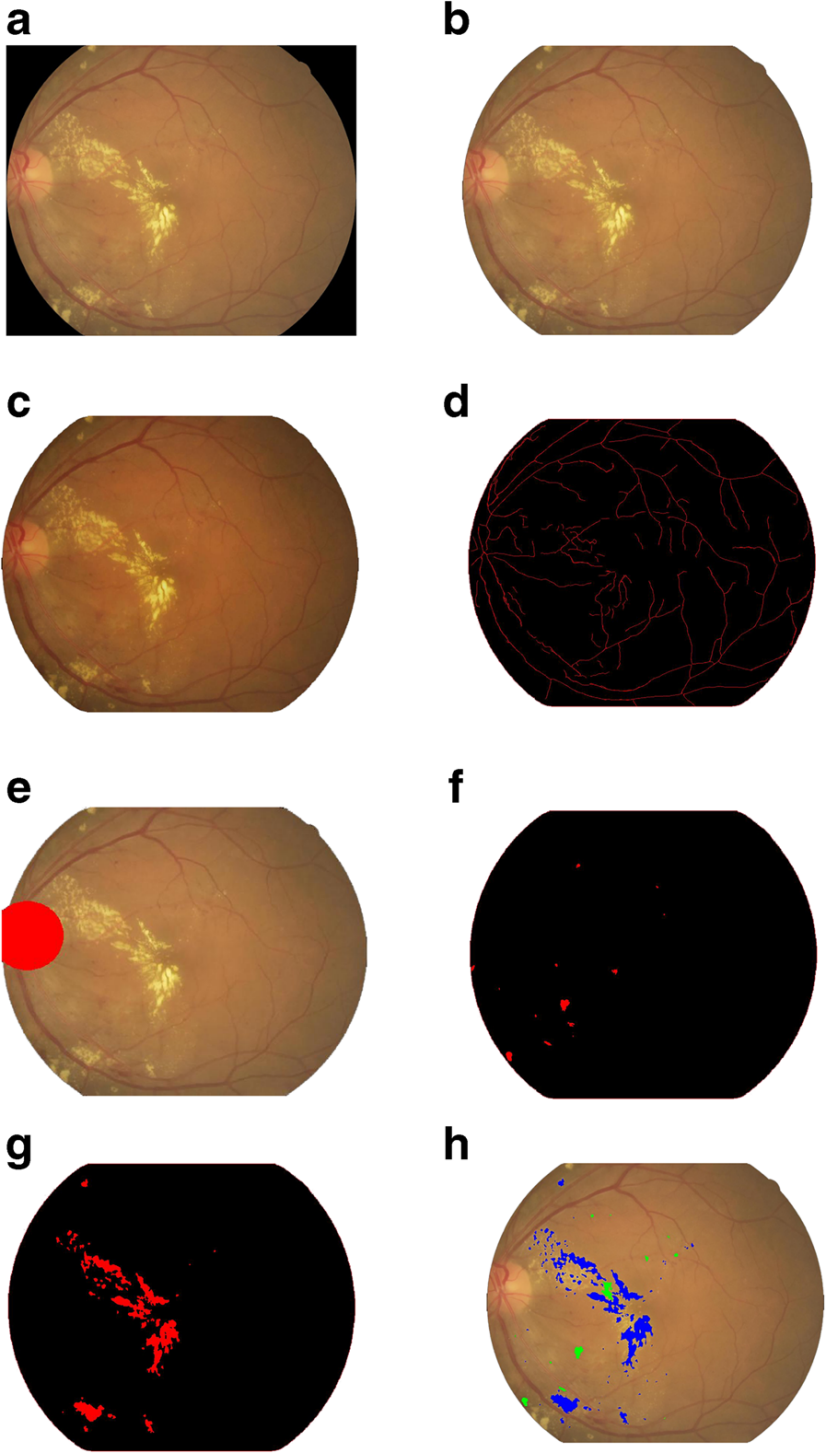
The image processing demonstration figures. **a** Original photograph, **b** Establish region of interest (ROI), extract the fundus image from original photograph, **c** Normalized processing, **d** Identify the fundus vascular borderline, **e** Identify optical disc, **f** Identify red lesions, **g** Identify bright lesions, **h** Highlight different lesions

**Fig. 3:**
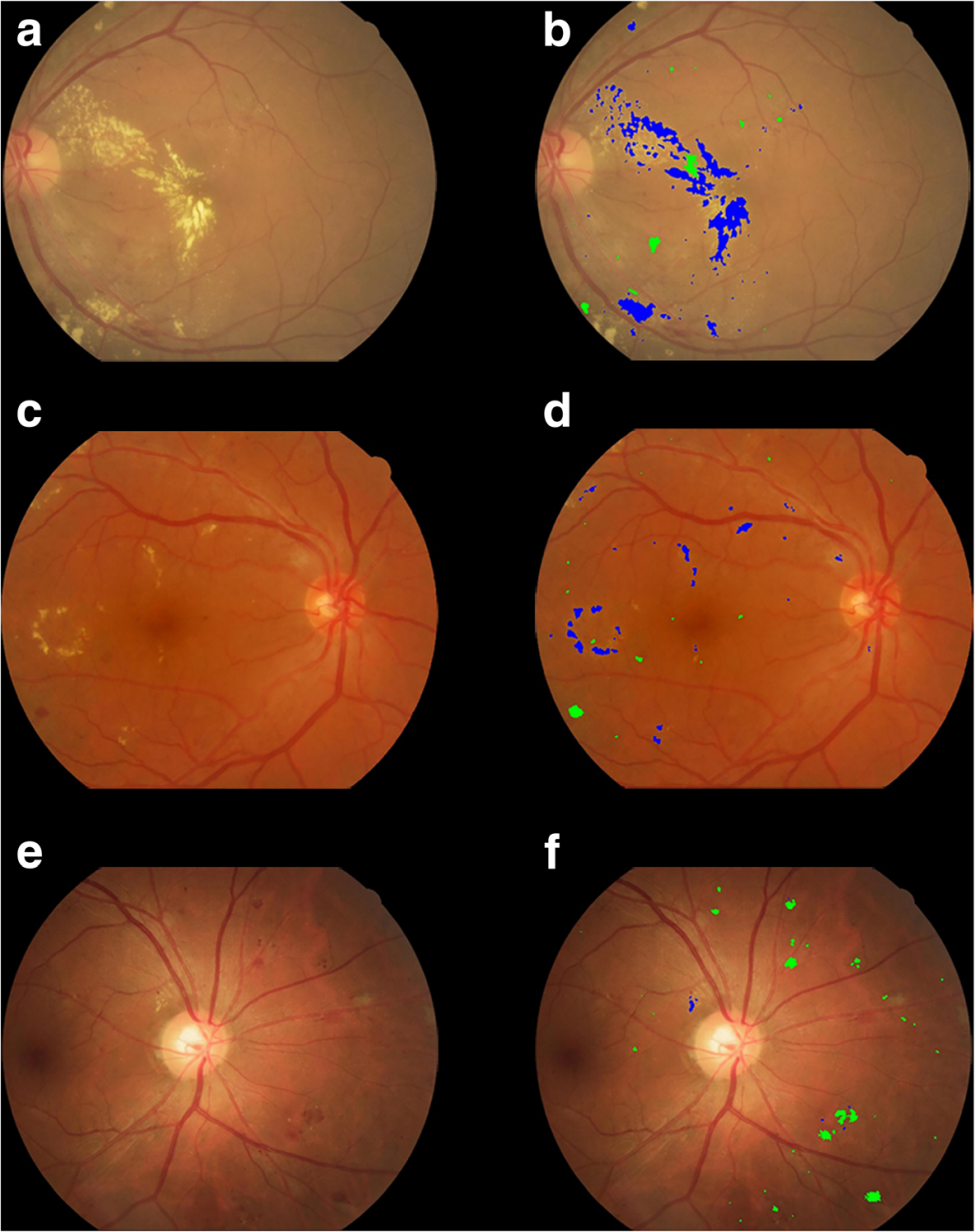
The fundus images of moderate NPDR (**a**, **c**, **e**). SmartEye marks hemorrhage and exudation in diabetic retinopathy with green and blue color (**b**, **d**, **f**). The area of haemorrhages in image **b**, **d**, **f** are 1528 pixels, 540 pixels, and 2387 pixels respectively; The area of exudation in image **b**, **d**, f are 12,214 pixels, 1785 pixels and 86 pixels respectively

**Fig. 4:**
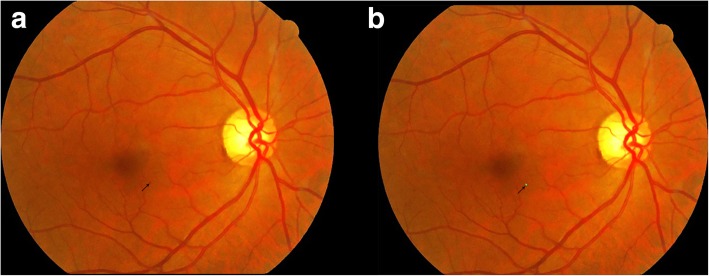
A fundus photograph of a patient with mild NPDR (**a**). A small microaneurysm which could be easily overlooked located in the parafoveal area. SmartEye recognized the lesion and marked it with green color (**b**)

Before the detection of fundus anatomical structure and lesions, the region of interest (ROI) is established by adaptive thresholding and template matching. Black background is removed. Because of differences in the resolution, color, luminance, and quality of images, all fundus photographs are adjusted according to the recommended reference value before evaluation, to ensure the precision of analysis results. The vascular outline, an important structure of the retina, should be marked correctly. SmartEye identifies the vascular borderline precisely through brightness threshold segmentation and color discrimination. The optical disc is recognized according to its brightness and shape, as well as the vascular direction. “Red lesions” such as microaneurysms, hemorrhages, and neovascularization are the critical characteristic lesions. Small red lesions are recognized on the basis of mathematical morphology, and large red lesions are identified through color discrimination. The shape, structure, color, and contrast of the focus are analyzed before a determination of a “red lesion” is made. Exudation and cotton-wool spots are two kinds of bright lesions in DR. SmartEye identifies such lesions through analyzing their shape, contrast, and color. Briefly, the image preprocessing process includes the following steps:establishing region of interest (ROI), normalized processing, identifying the vascular borderline, identifying and extracting the papilla disc before extracting DR lesions, to avoid its interference with the extraction of the lesion, identifying red lesions, and identifying bright lesions. The analytic steps and demonstration figures are shown in Fig. 1 and Fig. 2. SmartEye then marks the lesion area size at the pixel level (as shown in Fig. 3). Hemorrahge is marked with green, and exudation is marked with blue. Moreover, SmartEye is sensitive to “red lesions” and can identify such lesions that are difficult for the human eye to recognize (as shown in Fig. 4). The outcomes of different modules in SmartEye were combined, and the final diagnosis was acquired for further confirmation of DR. SmartEye was also able to calculate the hemorrhage/exudation lesion number and area. The lesion area was evaluated on the basis of pixel area.

### Data analysis

Sensitivity and specificity values were calculated for the entire group of participants and for subgroups with different stages of diabetes according to the fundus characteristics. Differences in sensitivity and specificity between the machine and clinician diagnosis were analyzed with McNemar’s test. The statistical analysis was performed in SPSS (version 19.0.0 for Mac; SPSS Inc., Chicago, IL, USA).

Sensitivity was defined as the proportion of diseased people correctly diagnosed, and specificity was defined as the proportion of non-diseased people correctly diagnosed. The rate of misdiagnosis was 1 - specificity, and the rate of missed diagnosis was 1 - sensitivity. The positive and negative predictive values are the proportions of positive and negative results in statistics and diagnostic tests that are true positive and true negative results, respectively.

A consistency check was used to determine the agreement in classification between machine and clinician diagnosis, expressed as a k value. The values for k were classified as follows: < 0.2, poor; 0.21 to 0.40, fair; 0.41 to 0.60, moderate; 0.61 to 0.80, good; and > 0.81, excellent. ROC analysis (sensitivity on the vertical axis and (1 – specificity) on the horizontal axis) was applied to evaluate the accuracy of SmartEye.

## Results

All 19,904 fundus images of 6013 patients with DM (23 to 97 years old (mean 69.65 ± 12.5 years) were included. All 19,904 fundus photographs were assessed by four diabetes retinopathy experts and SmartEye.

### Diagnostic accuracy

The diabetes eye classification from SmartEye was compared with the diagnoses made by clinicians (Table 1). In the sample of 19,904 images screened, 8369 (42.0%) patient images were disqualified for reading. The disqualified images usually had the features including fuzziness, large dark area, eyelash interference, overexposure to light or position deviation. Because that most of the participants were elderly people above 50 years-old, small pupil and various levels of lens opacity were commonly seen in these patients. Pupil dilation with cycloplegia was inconvenient because that only a few community healthcare centers having ophthalmology clinic in Shanghai and it is risky to use cycloplegia without ophthalmologists monitoring. 9266 (46.6%) patient fundus images appeared normal; 618 (3.1%) patients had mild nonproliferative diabetic retinopathy (NPDR); and 1190 (6.0%) patients had moderate NPDR. The number of patients with severe NPDR or above was 186 (0.9%). Among the 11,535 photos qualified for reading, the DR prevalence was 17.3%.

**Table 1:** Classification of outcomes of SmartEye compared with clinicians

Clinicians diagnosis	SmartEye diagnosis							
	DR0	DR1	DR2	DR3	PRP	Other diseases	Disqualification	Total
	N(%)	N(%)	N(%)	N(%)	N(%)	N(%)	N(%)	N(%)
No DR	5377 (58.0%)	290 (3.1%)	782 (8.4%)	223 (2.4%)	23 (0.2%)	74 (0.8%)	2497 (26.9%)	9266 (100.0%)
Mild NPDR	213 (34.5%)	152 (24.6%)	88 (14.2%)	5 (0.8%)	1 (0.2%)	7 (1.1%)	152 (24.6%)	618 (100.0%)
Moderate NPDR	176 (14.8%)	111 (9.3%)	583 (49.0%)	129 (10.8%)	3 (0.3%)	4 (0.3%)	184 (15.5%)	1190 (100.0%)
Severe NPDR	0 (0.0%)	0 (0.0%)	6 (8.3%)	65 (90.3%)	0 (0.0%)	0 (0.0%)	1 (1.4%)	72 (100.0%)
PDR	0 (0.0%)	0 (0.0%)	1 (8.3%)	8 (66.7%)	0 (0.0%)	0 (0.0%)	3 (25.0%)	12 (100.0%)
PRP	5 (4.9%)	5 (4.9%)	28 (27.5%)	20 (19.6%)	28 (27.5%)	0 (0.0%)	16 (15.7%)	102 (100.0%)
Other diseases	39 (14.2%)	10 (3.6%)	71 (25.8%)	86 (31.3%)	17 (6.2%)	1 (0.4%)	51 (18.5%)	275 (100.0%)
Disqualification	203 (2.4%)	50 (0.6%)	595 (7.1%)	391 (4.7%)	30 (0.4%)	3 (0.0%)	7097 (84.8%)	8369 (100.0%)
Total	6013 (30.2%)	618 (3.1%)	2154 (10.8%)	927 (4.7%)	102 (0.5%)	10,001 (50.2%)	89 (0.4%)	19,904 (100.0%)

**Fig. 5:**
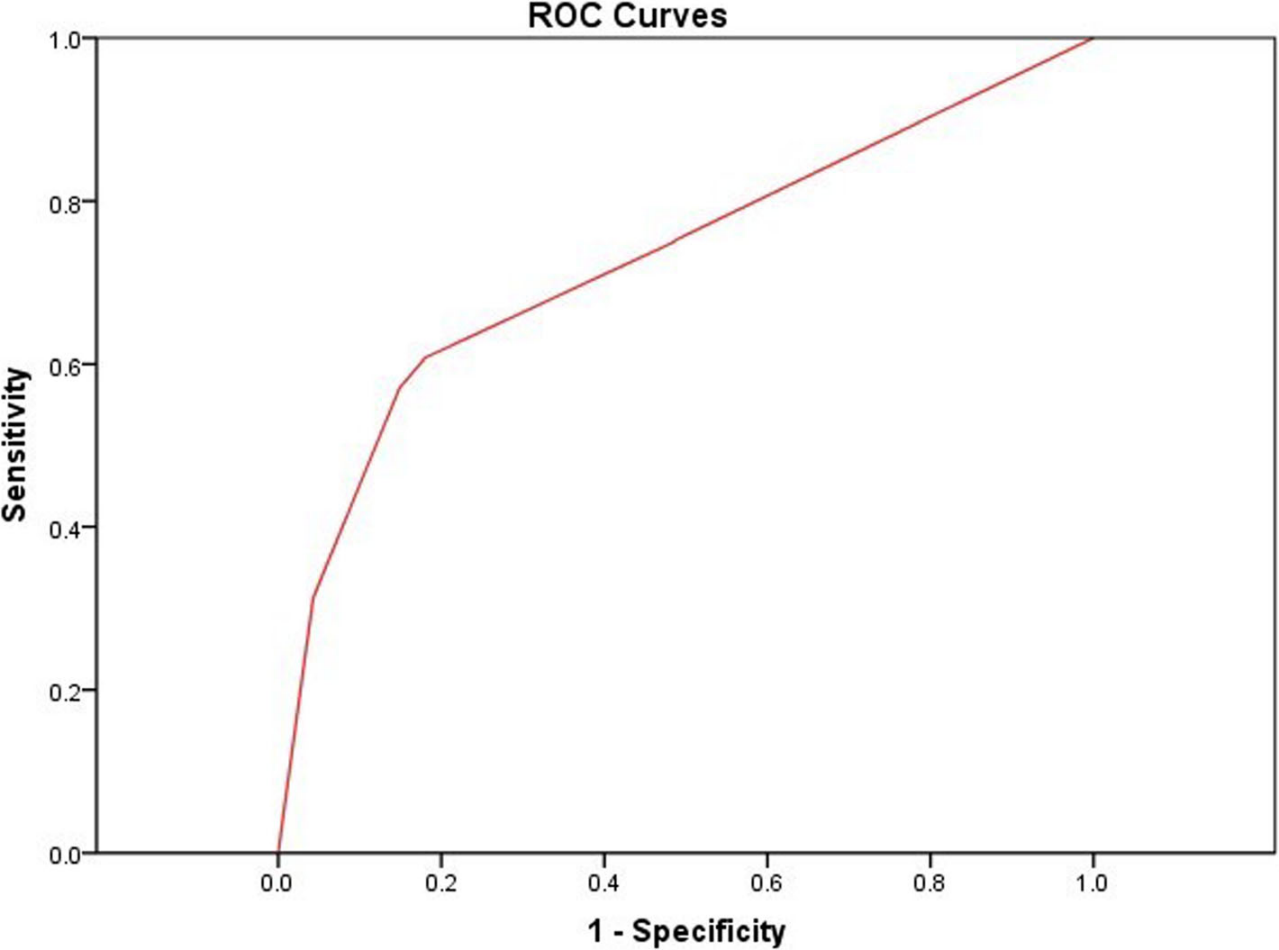
The ROC curve of SmartEye in the diagnosis of DR. The AUC was 0.73 (*p* < 0.001, 95%CI: 0.694–0.765)

**Fig. 6:**
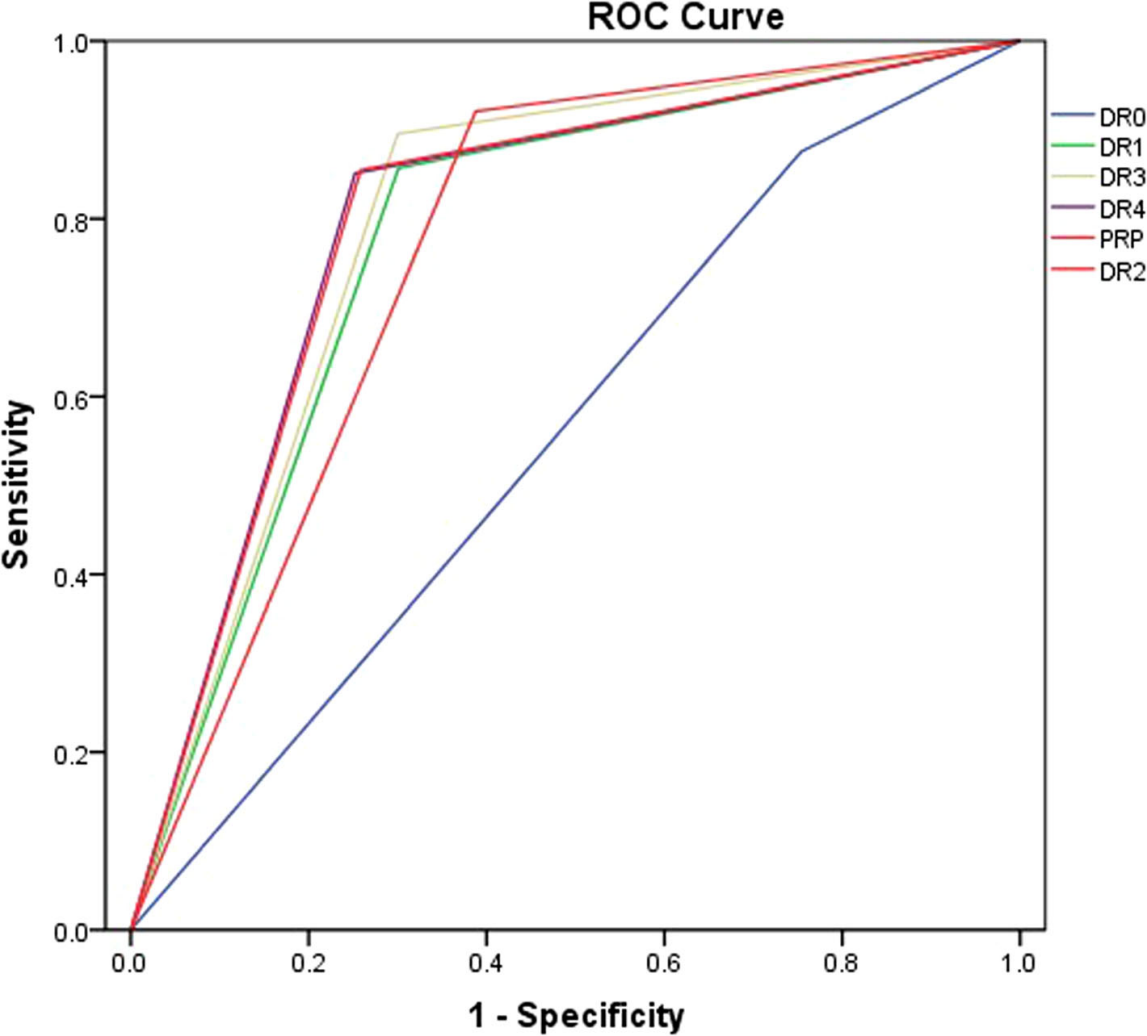
The ROC curve of SmartEye in the discrimination of DR on different stages. The AUC values were PRP, 0.80 (0.79, 0.81); PDR, 0.80 (0.79, 0.81); severe NPDR, 0.80 (0.79, 0.80); moderate NPDR, 0.77 (0.76, 0.77); and mild NPDR, 0.78 (0.77, 0.79), respectively

As shown in Table 2, the sensitivity of correctly classifying diabetic eyes stratified for no DR, mild NPDR, moderate NPDR, severe NPDR, PDR, and pan-retinal photocoagulation (PRP) were 86.19, 83.18, 88.64, 89.59, 85.02, and 84.96%, respectively. The specificity of the different DR stages was 63.07, 70.96, 64.16, 70.38, 74.79, and 74.33%, respectively. The diagnostic accuracy of severe NPDR was the highest (sensitivity, 89.59%; specificity, 70.38%). The kappa value increased with DR severity. The area under the ROC curve (AUC) of discriminating DR from normal fundus was 0.73 (*p* < 0.000, 95%CI: 0.694–0.765). For different stages of DR, the AUC values were PRP, 0.80 (0.79, 0.81); PDR, 0.80 (0.79, 0.81); severe NPDR, 0.80 (0.79, 0.80); moderate NPDR, 0.77 (0.76, 0.77); and mild NPDR, 0.78 (0.77, 0.79), respectively (Figs. 5 and 6). Thus, SmartEye had a high diagnostic accuracy. The AUC of discrimination of severe NPDR and above was greater than that of moderate NPDR and mild NPDR.

**Table 2:** Sensitivity and specificity for discriminating the classification of diabetic eyes by SmartEye

	Sensitivity (%)	Specificity (%)	The rate of missed diagnosis (%)	The rate of misdiagnosis (%)	Accuracy (%)	Positive Predictive Value (%)	Negative Predictive Value (%)	AUC (95% CI)	Kappa
No DR	86.19	63.07	13.81	36.93	83.50	94.65	37.58	0.56 (0.55,0.57)	0.38
Mild NPDR	83.18	70.96	16.82	29.04	76.45	70.01	83.81	0.78 (0.77,0.79)	0.53
Moderate NPDR	88.64	64.16	11.36	35.84	75.86	69.35	86.06	0.77 (0.76,0.77)	0.52
Severe NPDR	89.59	70.38	10.41	29.62	78.48	68.80	90.26	0.80 (0.79,0.80)	0.58
PDR	85.02	74.79	14.98	25.21	79.07	70.83	87.40	0.80 (0.79,0.81)	0.58
PRP	84.96	74.33	15.04	25.67	78.82	70.82	87.07	0.80 (0.79,0.81)	0.58

Several fundus images with high quality were chosen from different DR gradings, and the hemorrhage/exudation lesion number and area were calculated with SmartEye. The results are shown in Tables 3 and 4. Mild and moderate NPDR cases were included in group 1, severe NPDR cases were classified as group 2, and PDR cases were classified as group 3. There were significant differences in total hemorrhage area, hemorrhage number, maximum hemorrhage area, total exudation area, exudation number, and maximum exudation area among the three groups (Table 3). The sensitivity of pointing hemorrhage was from 88%(8%) to 96%(12%), and the sensitivity of pointing exudation was from 93%(17%) to 96%(11%) compared to the manual grading. The total hemorrhage area, maximum hemorrhage area, total exudation area, and maximum exudation area increased with the DR stage. The hemorrhage lesion number and exudation lesion number in group 2 (severe NPDR) were larger than those in group 3 (PDR). The hemorrhage lesion number and maximum hemorrhage area were different between each set of two groups. There was no difference in the total hemorrhage area in group 2 and group 3. The exudation lesion number increased significantly when DR progressed to severe NPDR. For the lesion quantification accuracy, lesion quantification was performed with SmartEye on these images for three times, and the results from three measurement were highly consistent (kappa value 1.0).

**Table 3:** ANOVA of total hemorrhage area, hemorrhage lesion number, maximum hemorrhage area, total exudation area, exudation lesion number, and maximum exudation area among different DR stages

Index	Frequency	Lesion area or number (^−^x ± s)	F	P
Total hemorrhage area			16.034	< 0.001
^a^Group 1	39	921.05 ± 1319.560		
^a^Group 2	29	15,218.59 ± 17,031.178		
^a^Group 3	23	16,479.22 ± 15,613.505		
Hemorrhage lesion number			37.100	< 0.001
Group 1	39	3.26 ± 2.702		
Group 2	29	26.62 ± 17.551		
Group 3	23	11.35 ± 9.384		
Maximum hemorrhage area			17.146	< 0.001
Group 1	39	636.03 ± 1021.743		
Group 2	29	3595.55 ± 3211.929		
Group 3	23	7133.70 ± 7550.703		
Total exudation area			10.553	0.001
Group 1	39	1496.46 ± 2699.786		
Group 2	29	6589.90 ± 10,347.651		
Group 3	23	15,062.87 ± 18,862.476		
Exudation lesion number			5.552	0.005
Group 1	39	6.00 ± 10.665		
Group 2	29	20.66 ± 26.203		
Group 3	23	11.96 ± 14.729		
Maximum exudation area			15.256	< 0.001
Group 1	39	578.87 ± 1363.809		
Group 2	29	1622.69 ± 3186.810		
Group 3	23	7084.78 ± 8306.680		

^a^ Group 1, mild NPDR and moderate NPDR; group 2, severe NPDR; group 3, PDR

**Table 4:** Comparison of total hemorrhage area, hemorrhage lesion number, maximum hemorrhage area, total exudation area, exudation lesion number, and maximum exudation area among different DR stages

Dependent variable	Group		Mean difference	Standard error	P	95% Confidence Interval	
						Lower limit	Upper limit
Hemorrhage area	^a^Group 1	^a^Group 2	−14,297.535*	3042.766	< 0.001	−20,344.39	− 8250.68
		^a^Group 3	−15,558.166*	3262.459	< 0.001	−22,041.62	− 9074.71
	Group 2	Group 1	14,297.535*	3042.766	< 0.001	8250.68	20,344.39
		Group 3	− 1260.631	3464.848	.717	− 8146.29	5625.03
	Group 3	Group 1	15,558.166*	3262.459	< 0.001	9074.71	22,041.62
		Group 2	1260.631	3464.848	.717	− 5625.03	8146.29
Hemorrhage lesion number	Group 1	Group 2	−23.364*	2.721	< 0.001	−28.77	−17.96
		Group 3	−8.091*	2.918	.007	−13.89	−2.29
	Group 2	Group 1	23.364*	2.721	< 0.001	17.96	28.77
		Group 3	15.273*	3.099	< 0.001	9.11	21.43
	Group 3	Group 1	8.091*	2.918	.007	2.29	13.89
		Group 2	−15.273*	3.099	< 0.001	−21.43	−9.11
Maximum hemorrhage area	Group 1	Group 2	− 2959.526*	1039.915	.006	− 5026.14	− 892.91
		Group 3	− 6497.670*	1114.998	< 0.001	− 8713.49	− 4281.85
	Group 2	Group 1	2959.526*	1039.915	.006	892.91	5026.14
		Group 3	− 3538.144*	1184.168	.004	− 5891.43	− 1184.86
	Group 3	Group 1	6497.670*	1114.998	< 0.001	4281.85	8713.49
		Group 2	3538.144*	1184.168	.004	1184.86	5891.43
Exudation area	Group 1	Group 2	− 5093.435	2754.178	.068	−10,566.79	379.92
		Group 3	−13,566.408*	2953.034	< 0.001	−19,434.94	− 7697.87
	Group 2	Group 1	5093.435	2754.178	.068	− 379.92	10,566.79
		Group 3	− 8472.973*	3136.228	.008	−14,705.57	− 2240.38
	Group 3	Group 1	13,566.408*	2953.034	< 0.001	7697.87	19,434.94
		Group 2	8472.973*	3136.228	.008	2240.38	14,705.57
Exudation lesion number	Group 1	Group 2	−14.655*	4.399	.001	−23.40	−5.91
		Group 3	−5.957	4.716	.210	−15.33	3.42
	Group 2	Group 1	14.655*	4.399	.001	5.91	23.40
		Group 3	8.699	5.009	.086	−1.26	18.65
	Group 3	Group 1	5.957	4.716	.210	−3.42	15.33
		Group 2	−8.699	5.009	.086	−18.65	1.26
Maximum exudation area	Group 1	Group 2	− 1043.818	1131.248	.359	− 3291.94	1204.30
		Group 3	− 6505.911*	1212.926	< 0.001	− 8916.35	− 4095.48
	Group 2	Group 1	1043.818	1131.248	.359	− 1204.30	3291.94
		Group 3	− 5462.093*	1288.171	< 0.001	− 8022.06	− 2902.12
	Group 3	Group 1	6505.911*	1212.926	< 0.001	4095.48	8916.35
		Group 2	5462.093*	1288.171	< 0.001	2902.12	8022.06

*p < 0.05

^a^ Group 1, mild NPDR and moderate NPDR; group 2, severe NPDR; group 3, PDR

## Discussion

This study demonstrates that SmartEye has the good potential of big-scale DR screening in communities, which is a challenge in countries with huge population and low-ratio of ophthalmologists to population. SmartEye is a computer assisted diagnosis system with a lesion size quantification function, which was designed to discriminate DR from normal fundus and to classify DR stages according to the International Diabetic Retinopathy classification [15]. In the present study, we compared the diagnosis consistency between SmartEye and clinicians based on the images taken with nonmydriatic cameras in community healthcare centers. In an upcoming study, we will analyze the lesion quantification function of SmartEye in different stages of DR.

SmartEye’s diagnostic sensitivity was higher than its specificity. Similarly to results from other studies, SmartEye performed better in the diagnosis of severe NPDR or above, possibly because the number of lesions increases markedly after progression to severe NPDR and PDR, thus aiding in differentiation from normal fundus. Photocoagulation spots were also distinctive lesions in patients who had undergone pan-retinal photocoagulation (PRP). All fundus images were collected from patients without cycloplegia treatment, in contrast to the high-quality images used in other studies. Determining proliferative changes without using fluorescence fundus angiography examination and with only nonmydriatic fundus examination is extremely difficult. However, because of the ophthalmological medical resource limitation in community healthcare center, only basic fundus photos screening could be performed in DM patients. For human grading, three ophthalmologists grade the fundus images independently, then a consensus meeting was held to discuss images without initial agreement. Although the fundus images were taken with natural pupil size, SmartEye still had good performance in DR identification and grading. With the current widespread use of non-mydriatic fundus cameras [16–18], the SmartEye system may have broad application prospects.

Several studies have recently reported the application of an automated DR-screening system in the discrimination and classification of DR. SmartEye has several advantages over those diagnosis systems [19–21], as follows:

1. In early stages of diabetic retinopathy, the lesion area is small, and the characteristics are not obvious. Discriminating these lesion areas accurately is difficult, especially in older non-mydriatic patients whose lens opacity may influence the image resolution. Whereas a clinician’s eye might take a long time to identify the lesion, SmartEye is able to recognize the characteristic lesion quickly and screen out early DR with high efficiency. In this case, patients with early diabetic retinopathy would have the opportunity for further evaluation and treatment in a timely manner.
2. SmartEye not only identifies the lesion location but also marks the lesion size at the pixel level. This function has not been reported in other studies of DR diagnosis systems, such as ARIAS and CAD. Those systems might help doctors understand whether patients have DR but cannot help them identify the lesions of the disease. By quantifying the lesion area, SmartEye may allow doctors to determine the severity of the diabetic retinopathy. In addition, SmartEye may allow doctors to observe disease progression and perform follow up regarding the DR status of patients.

All images used to evaluate SmartEye’s discrimination ability in this study were acquired during the SDES study, in which well-trained community healthcare professionals took fundus photographs with a non-mydriatic fundus cameras. The images are different from other study images taken from public databases. The AUC for identifying mild NPDR was 0.78, and the AUC for diagnosing severe NPDR and above reached 0.80. The present study results should provide a foundation for further studies and community DR screening programs using non-mydriatic fundus cameras.

DR lesion quantitative analysis is a function of SmartEye. Hemorrhage and exudation were the major recognizable lesions in DR. SmartEye measured the lesions by number of pixels. The lesion size increased markedly with the progression of DR stage, in accordance with the clinical features of DR. The hemorrhage and exudation lesion number decreased from severe NPDR to PDR, possibly because when DR progressed to PDR, previous lesions enlarged and fused together. Consequently the lesion number decreased, but the lesion size was enlarged. SmartEye quantitative analysis may be an innovative and promising method for DR diagnosis and grading. Further evaluation and follow up studies are necessary.

For quantitative analysis of lesion areas, SmartEye is able to extract the lesion area and introduce pixel level labels. However, in actual clinical practice, it could be used only for auxiliary diagnosis, and further clinical verification would still be needed. In the future, SmartEye is expected to include further improvements in quantitative extraction, and it may aid in establishing quantitative criteria for disease grades.

In further studies, the comparison between SmartEye and other systems applying deep-learning technique will be performed. Regarding the advantages of SmartEye compared to deep-learning based DR diagnosis systems, SmartEye screens DR based on lesions of DR, which does not require a large number of samples for training. Certain deep-learning based DR diagnosis systems are capable to simply label abnormal characteristics of the fundus, but it may not achieve accurate labeling, especially for the labeling of tiny lesions. In addition, deep-learning based on DR diagnosis systems usually cannot quantify the lesion, thus the changes of DR might not be accurately represented [22, 23].

## Conclusions

Like most other systems, SmartEye was designed to identify and screen for a single disease. On the basis of its image identification ability and lesion quantification function, SmartEye may have wide application potential in the diagnosis of other ophthalmic diseases. We will continue to do research and development work on this system to enrich its clinical value in the near future.

## Data Availability

The datasets generated during and/or analysed during the current study are available from the corresponding author upon reasonable request.

## Abbreviations

DM: Diabetes mellitus
DR: Diabetic retinopathy
HIS: The Indian Health Service
JVN: Joslin Vision Network
NHS DESP: The UK National Health Service Diabetic Eye Screening Program
NPDR: Non-proliferative diabetic retinopathy
PDR: Proliferative diabetic retinopathy
PRP: Pan-retinal photocoagulation
ROI: Region of interest
SDES: The Shanghai Diabetic Eye Study in Diabetics
SEDPTC: Shanghai Eye Disease Prevention & Treatment Center
